# Decisions for Others Become Less Impulsive the Further Away They Are on the Family Tree

**DOI:** 10.1371/journal.pone.0049479

**Published:** 2012-11-28

**Authors:** Fenja V. Ziegler, Richard J. Tunney

**Affiliations:** 1 School of Psychology, University of Lincoln, Lincoln, United Kingdom; 2 School of Psychology, University of Nottingham, Nottingham, United Kingdom; Institut Pluridisciplinaire Hubert Curien, France

## Abstract

**Background:**

People tend to prefer a smaller immediate reward to a larger but delayed reward. Although this discounting of future rewards is often associated with impulsivity, it is not necessarily irrational. Instead it has been suggested that it reflects the decision maker’s greater interest in the ‘me now’ than the ‘me in 10 years’, such that the concern for our future self is about the same as for someone else who is close to us.

**Methodology/Principal Findings:**

To investigate this we used a delay-discounting task to compare discount functions for choices that people would make for themselves against decisions that they think that other people should make, e.g. to accept $500 now or $1000 next week. The psychological distance of the hypothetical beneficiaries was manipulated in terms of the genetic coefficient of relatedness ranging from zero (e.g. a stranger, or unrelated close friend), .125 (e.g. a cousin), .25 (e.g. a nephew or niece), to .5 (parent or sibling).

**Conclusions/Significance:**

The observed discount functions were steeper (i.e. more impulsive) for choices in which the decision-maker was the beneficiary than for all other beneficiaries. Impulsiveness of decisions declined systematically with the distance of the beneficiary from the decision-maker. The data are discussed with reference to the implusivity and interpersonal empathy gaps in decision-making.

## Introduction

Each day people have to make decisions, weighing up options and choices both in relatively small and trivial matters and in those with potentially life-changing consequences. The last 30 years of decision-making research have rendered the idea that decision-makers are primarily guided by rational processes, embodied in the idea of homo economicus, little more than a straw man [1].

There are numerous examples which demonstrate the prominent role of emotions in the decision making process. For example, in decisions which present the choice between an immediate reward and a later reward, the choice alternative which is only available after a delay is worth less than the same amount available now. The later alternative is discounted by time. This is not only true when the choice options are of the same nominal value, but also when a smaller but immediate reward is pitched against a later but larger reward. Depending on the size of difference between the rewards and the length of the delay, people often show a preference for the smaller immediate reward and this seems to be driven by the immediate gratification it brings. The preference for the smaller reward will disappear when it is not immediately but only relatively sooner available than the later larger reward, depending upon how impulsive the decision-maker is. For example, one decision maker may prefer $10 now to $20 dollars in 2 weeks, but when the time delay is moved into the future so that the choices are $10 in 1 year or $20 in 1 year and 2 weeks, then decision makers prefer the later, larger reward. A different person (or indeed one making a different decision) may only change their preference after a much longer delay. This strengthens the interpretation that these impulsive decisions are driven by seeking immediate gratification, because when this is not available through the earlier reward, the preference is for waiting for the larger later reward. Whilst this decision making is impulsive it may not necessarily be irrational, but a reflection of decision makers caring more about their present self (me now) than a future self (me in 10 years) [2], [3]. There are individual differences in decision makers’ impulsiveness [4]. Interestingly, Mischel and colleagues [5]–[7] found that children’s ability to forego a small reward (1 marshmallow) for a larger later reward (2 marshmallows in 5 minutes) predicted their later scholastic and life success [8]–[10].

Whilst not a rational process decision makers nonetheless follow predictable patterns in their decisions and much research has been dedicated to understanding the exact processes underpinning these decisions, with a particular focus on decisions made for the decision-makers themselves. Many of the daily decisions made are indeed for ourselves, however, a sizeable number of decisions are actually made for others in a number of ways. For example, decision outcomes may affect both the decision maker and others or sometimes only others, we seek and give advice when faced with decisions, and particularly in medical and financial contexts, decisions are made for others by proxy and the tacit assumption in these ‘other-decisions’ is that decision-makers are able to accurately predict the choice or outcome preference of the target of the decision. The emotional component of decision-making, however, presents sources of error, which can affect ‘other-decisions’. The risk-as-feeling hypothesis [11] explains people’s choices as partly driven by experiencing positive or negative emotions in relation to risk or uncertainty, where positive emotions lead to risk seeking behaviour (e.g. buying a lottery ticket) and negative emotions to risk aversion (e.g. buying insurance). However in making decisions for others there may be a hot-cold-empathy gap [12], which leads us to believe that others have more muted emotional reactions to risk and uncertainty than they actually do and that these reactions influence others’ choices less than they do. We would thus expect decisions made for others to be systematically less influenced by emotional responses than decisions for self. However, the results from a number of empirical studies do not present a clear pattern of difference in self and other decisions. Some studies show the expected hot-cold empathy gap: decision-makers were more likely to choose an immediate reward for themselves but delayed reward for other people [3], receiving immediate rewards showed greater activation in the dopaminergic reward system for self but not other decisions [13], give more risk seeking advice about romantic relationships compared to self-decisions [14] and predict less risk seeking and less risk averse behaviour in others compared to self [15]. Other studies however find no difference in self-other decisions in risk taking [16], waiting time decisions [17], predicted choice of another on a financial task relative to self [18] and Wray and Stone [19] comment that there are generally no self-other differences in risky monetary situations. Given the importance of many decisions made for others and the lack of accuracy in many proxy decisions, for example, most proxy medical end-of-life decisions do not reflect the preference of the patient but those of the decisions maker [20], it is crucial to understand the circumstances under which other-decisions are different from self-decisions and the source of this difference. Identifying the source of difference is the first step in constructing a model which allows for more accurate decision making.

One important aspect of difference in studying decisions made for others is the identity of ‘the other’ and in some studies this identity has been found to make a difference (e.g. [18]) but without giving rise to a predictive pattern. Jones and Rachlin [21] found that social distance from the decision-maker to the target of the decision systematically influenced how much money the decision maker was willing to forego in order to also give a reward to another person. This process of social discounting varied systematically with the perceived closeness to the other and was greatest for relatives or close friends and smallest for mere acquaintances. Jones and Rachlin [21], [22] stress that social discounting is not solely due to genetic overlap, but we propose that genetic overlap as measured by degree of kinship is a simple and systematic way to model social distance. Genetic kinship is expressed by the relatedness co-efficient *r*, with 1 describing the closest relationship (self) and decreasing numbers reflect greater familial distance, e.g., *r = .5* includes parents and siblings, *r = .25* includes grandparents, uncles, aunts, nieces and nephews, *r = .125* includes cousins and so forth [23], [24].

Making decisions in a delay-discounting paradigm requires participants to make a number of decisions in which they trade-off the gratification of the smaller but immediate reward (e.g. $700 now) against a fixed larger but delayed reward (e.g. $1000 in 2 weeks). The switch towards the larger, delayed reward depends on the relative size of the smaller reward (a proportionally smaller reward is less attractive) and the temporal distance of the larger reward (when in the future is it available). The draw of the smaller reward is the experience of immediate gratification and, based on the predictions of the hot-cold empathy gap, we predict that participants are less impulsive when making decisions for others overall. Furthermore, if the identity of the ‘other’ as a target of decisions is important then we expect that the degree of relatedness as a model of social distance will have a systematic influence on how impulsive decisions are made on their behalf compared to decisions participants make for themselves.

## Materials and Methods

### Ethics Statement

The study was approved by the ethics committees of the School of Psychology at the University of Nottingham and the School of Psychology at the University of Lincoln. All of the participants provided written informed consent prior to their participation.

### Participants

Seventy undergraduate students from the universities of Nottingham and Lincoln volunteered to take part in the experiment. Sixty-one were female and 9 were male. The age group ranged from 18 to 21 (M 18.66, SD 0.90). The number of siblings, participants had, ranged from 0 to 4 (M 1, SD 0.96).

### Stimuli

The experiment was run in Psychopy [25]. The stimuli consisted of 450 choices between an immediate outcome and a larger delayed outcome. Each item took the form:

Should [*r*] accept [*v*] now or wait for $1000 in [*d*]?

We manipulated social distance using Wright’s [2] co-efficient of relationship (*r*). This resulted in 5 categories of relationship: r = 1 “you”, r = 0.5 “your mother”, “your father”, “your brother” or “your sister”; r = 0.25 “your aunt”, “your uncle”, “your nephew”, or “your niece”; r = 0.125 “your cousin”; and r = 0 “your best friend” or “a stranger”. The immediate values (a) were $1, $10, $20, $60, $100, $150, $250, $400, $500, $600, $750, $850, $900, $950, or $1000. The delayed value (*a*) was fixed at $1000, but the delay time (*d*) could be “7-days”, “14-days”, “1-month”, “6-months”, “1-year”, or “5-years”.

On each trial the participant indicated their choice by pressing one of two keys marked “now” or “later”. After this the stimulus disappeared and the next trial followed a 1.5sec inter-trial interval. The order of presentation of the trials was fully randomized for each participant so no two participants saw the same sequence of trials [26]. The experiment was self-paced but took on average 45 minutes to complete.

## Results

Impulsivity in inter-temporal choice is best described by a hyperbolic function [27] in which the subjective discounted value (*v*) of a sum (*a*) is discounted as a function (*k*) of the delay (*d*) before the sum (*a*) can be received [28].
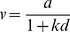



Small value of *k* indicates self-control in the sense that the decision-maker is willing to wait longer to receive the reward. Larger values of *k* indicate impulsivity.

**Figure 1:**
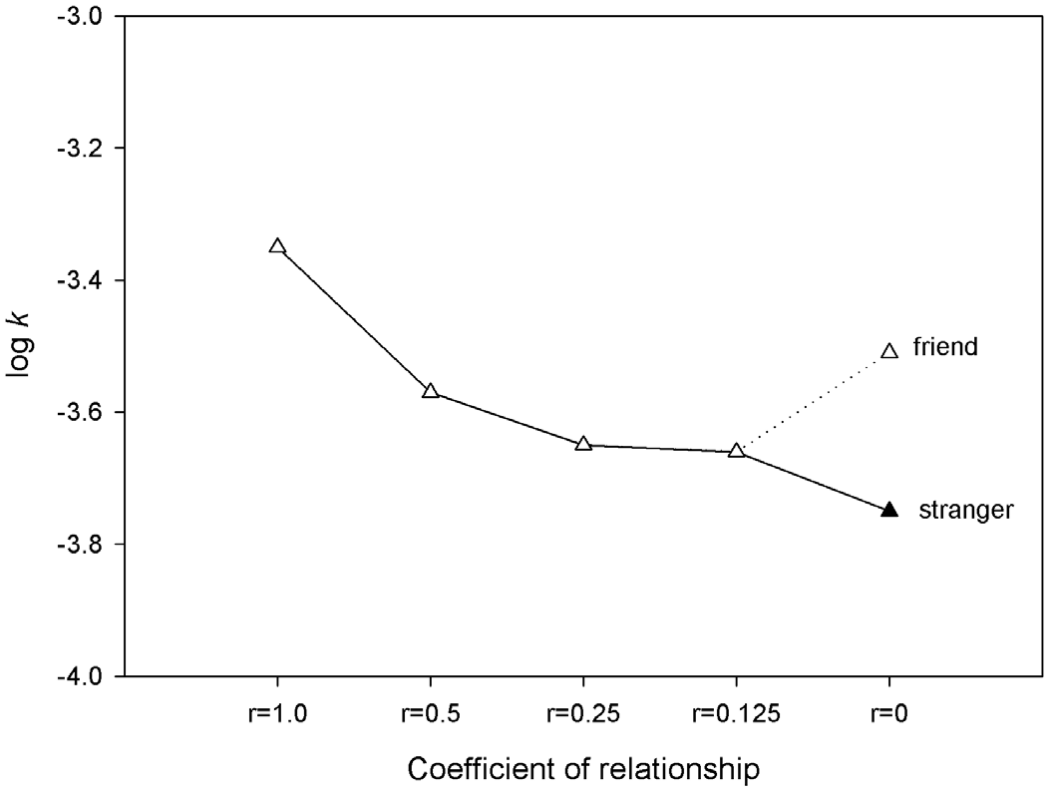
Average discount rates for each level of social distance. The participant has a relationship of 1 with themselves which decreases systematically with degree of relatedness *r = *.5 includes parents and siblings; *r* = .25 includes aunts, uncles, nephews and nieces; *r* = 125 includes cousins. Importantly, at 0 relatedness someone could be a stranger or a close friend.

**Figure 2:**
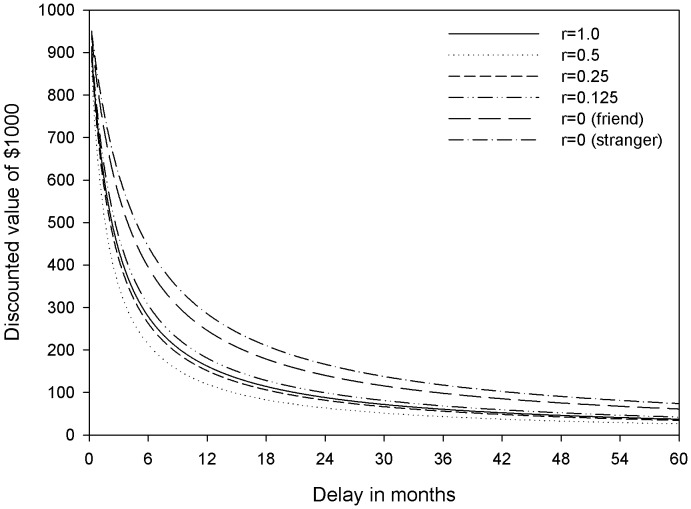
Discounted subjective values of $1000 as a function of r and delay.

The dependent variable *k* was estimated separately for each participant and for each level of social distance. To do so, we first found the average value (*v*) of the delayed amount (a = $1000) either side of the crossover point at which the participants switched from preferring the larger later reward for the smaller sooner reward. We then used a nonlinear regression to estimate the best fitting value of *k* to describe the discount function across the different delayed values. This resulted in 5 discount parameters, one for each social distance, for each participant. Since these were highly skewed these parameters were then log-transformed. The average discount rates for each level of social distance are shown in Figure 1. Figure 2 shows the effect of these different discount rates in the subjective values of $1000 as a function of r and delay. The log(*­k*) values were entered into a repeated-measures ANOVA with social distance as the within-subjects factor with 5 levels (*r* = 1, *r* = .5, *r* = .25, *r* = .125 and *r* = 0). For this first analysis the discount parameters for *r* = 0 were computed over decisions made on behalf of both best friends and strangers.

The results showed a reliable quadratic trend of discount rates by social distance *F*(1, 68) = 5.71, *MSE* = 0.92, *p*<.05, 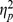 = .08, but did not show a linear trend *F*(1,69) <1.0. Because we wanted to examine the effects of familiarity and the potential effects of empathy we next re-ran the analysis replacing the average *r* = 0 level with *r* = 0 computed just over the best friend category. This also revealed a reliable quadratic trend *F*(1,68) = 4.48, *MSE* = 0.87, *p*<.05, 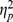 = 0.06, but no linear trend *F*(1,68) <1.0. This suggests that as social distance increases the participants made less impulsive decisions relative to themselves except when the recipient was their best friend. To confirm this we ran the analysis with r = 0 computed over the stranger category. This revealed a reliable linear trend *F*(1,68) = 6.81, *MSE* = 0.82, *p*<.01, 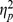 = 0.91, but no quadratic component *F*(1,69) <1.0. Figure 1 shows a clear difference in discount functions for unrelated best friends for whom our decisions are similar in impulsivity to our own, compared with unrelated strangers for whom our decisions are less impulsive than our more distant relations. This suggests that we become increasingly impartial about decisions as our familiarity or distance with a recipient increases. That is, we think that other people should make impartial decisions, but the more familiar we are with them, or the closer in social distance, the less self-controlled we think other people ought to be.

## Discussion

The experiment reported here examined whether social distance affects the impulsiveness of decisions that people make on behalf of others. We manipulated social distance in terms of familial relationships that can be conveniently measured numerically in terms of the coefficient of relationship. Although we make no claim specifically about how kin selection might influence decision-making, we assume that there is a positive relationship between familiarity and the coefficient of relationship. In terms of models of decision-making this familiarity involves varying degrees of empathy. It follows that the closer our relationship with another the greater our empathy with that person. A good deal of research suggests that decision-making is often less than optimal because our emotions influence the subjective value or probability of an outcome. This is apparent in impulsivity and in the discounting of future rewards and losses. Since we have empathetic links with other people it follows that we are likely to have varying degrees of feeling for their potential outcomes. Indeed the results of our experiment confirm that the closer our relationship with another person the closer our discount function for decisions that we might make for them is to our own.

The results show that the underlying decision making process is capable of making rational controlled decisions in the sense that the discount rates for decision made for unrelated people are far lower than those that we make ourselves [18]. These are cold appraisals of the outcomes presumably because we have no connection with strangers [18]. That is, when there is an interpersonal empathy gap, emotions influence the decision less and the outcome is more optimal in the economic sense. However, the discount functions for hypothetical best friends are similar to our first-degree relatives, presumably because although the coefficient of relationship is zero, our familiarity and closeness to our best friends is high. We expect this effect to be limited to the reward frame because we would not expect an individual to make a decision that would cause their close relatives, or best friend, pain or loss. For example, a parent might encourage their offspring to save money, but tend not to encourage them to engage in self-destructive behaviors such as smoking, even when they themselves might smoke.

The burgeoning literature on surrogate-decision making shows inconclusive effects of changing the target of decision from ‘self’ to ‘other’ [1], [3], [14], [19]. Here we show the first demonstration that the identity of the beneficiary has a systematic effect on the decision-makers’ impulsivity. This has important implications for understanding the processes underlying decisions made for others and the interpretation of existing research in the area, because it suggests that asking decision-makers about an “other” is not a meaningful category, if the perceived closeness to the “other” systematically changes the emotional biases observed in decisions. This may explain the disparity in the findings of the existing proxy decision making literature, which does not consistently report differences between self and other decisions. It implies that in future research the identity of the ‘other’ needs to be specified and comparisons between studies need to focus more on the identity of the ‘other’ and not presume that findings can be extrapolated from one specific group of beneficiaries to another.

The surrogate-decision making literature must consider the relationship between the decision-maker and the beneficiaries of those decisions, because the precise nature of the relationship between the self and other can affect the optimality of those decisions. For example, surrogate medical decisions if made by a close relative of the patient may reflect their own preferences rather than the preferences of the patient [16]. If so, this would further question the validity of the substituted judgment as the gold standard in medical surrogate decision making [29], since this model assumes that the decision maker can accurately state the patient’s wishes independently and irrespective of their own. At first glance it looks as though the most optimal decisions are made by people who are unrelated to the recipient, but this neglects the emotional appraisal of the decision outcome and only reflects the economic outcome. Thus a physician might be best placed to make a substituted judgment on behalf of a patient but this may well be a different judgment than the one made by a close relative. It is a value judgment whether the economic benefit should outweigh the perceived personal satisfaction of the decision outcome.

Future research should investigate whether perceived social closeness, as modeled by relatedness, affects the importance we ascribe to decision-outcomes which are based on emotional aspects compared to impartial aspects of increased utilities.

## References

[1] Kahneman D (2011) Thinking fast and slow. London: Penguin.

[2] Parfit D (1971) Personal identity. The Philosophical Review 80: 3–27.

[3] Pronin E, Olivola CY, Kennedy KA (2008) Doing unto future selves as you would do unto others: Psychological distance and decision making. Personality and Social Psychology Bulletin 34: 224–236. 18156588 10.1177/0146167207310023

[4] Brown SM, Manuck SB, Flory JD, Hariri AR (2006) Neural basis of individual differences in impulsivity: Contributions of corticolimbic circuits for behavioral arousal and control. Emotion 6: 239–245. 16768556 10.1037/1528-3542.6.2.239

[5] Mischel W, Metzner R (1962) Preference for delayed reward as a function of age, intelligence, and length of delay interval. Journal of Abnormal and Social Psychology 64: 425–431. 14474526 10.1037/h0045046

[6] Mischel W, Shoda Y, Rodriguez MI (1989) In person perception: Effects of situation, behavior relations on dispositional judgments. Journal of Personality and Social Psychology 56: 41–53. 2926616 10.1037//0022-3514.56.1.41

[7] Metcalfe J, Mischel W (1999) A hot/cool system analysis of delay of gratification: Dynamics of willpower. Psychological Review 106: 3–19. 10197361 10.1037/0033-295x.106.1.3

[8] Shoda Y, Mischel W, Peake PK (1990) Predicting adolescent cognitive and social competence from preschool delay of gratification: Identifying diagnostic conditions. Developmental Psychology 26: 978–986.

[9] Eigsti IM, Zayas V, Mischel W, Shoda Y, Ayduk O, et al (2006) Predicting cognitive control from preschool to late adolescence and young adulthood. Psychological Science 17: 478–484. 16771797 10.1111/j.1467-9280.2006.01732.x

[10] Casey BJ, Somerville LH, Gotlib IH, Ayduk O, Franklin NT, et al (2011) Behavioral and neural correlates of delay of gratification 40 years later. Proceedings of the National Academy of Sciences of the United States of America 108: 14998–15003. 21876169 10.1073/pnas.1108561108 PMC3169162

[11] Loewenstein GF, Weber EU, Hsee CK, Welch ES (2001) Risk as feelings. Psychological Bulletin 127: 267–286. 11316014 10.1037/0033-2909.127.2.267

[12] Loewenstein GF (1996) Out of control: Visceral influences on behavior. Oraganizational Behavior and Human Decision Processes 65: 272–292.

[13] Albrecht K, Volz KG, Sutter M, Laibson DI, von Cramon DY (2011) What is for me is not for you: Brain correlates of intertemporal choice for self and other. Social Cognitive and Affective Neuroscience 6: 218–225. 20529885 10.1093/scan/nsq046 PMC3073390

[14] Beisswanger AH, Stone ER, Hupp JM, Allgaier L (2003) Risk taking in relationships: Differences in deciding for oneself versus for a friend. Basic and Applied Social Psychology 25: 121–135.

[15] Faro D, Rottenstreich Y (2006) Affect, empathy and regressive mispredictions of others’ preferences under risk. Management Science 52: 529–541.

[16] Benjamin AM, Robbins SJ (2007) The role of framing effects in performance on the balloon analogue risk task (BART). Personality and Individual Differences 43: 221–230.

[17] Krishnamurthy P, Kumar P (2002) Self-other discrepancies in waiting time decisions. Organizational Behaviour and Human Decision Processes 87: 207–226.

[18] Hsee CK, Weber EU (1997) A fundamental prediction error: Self-others discrepancies in risk preference. Journal of Experimental Psychology: General 126: 45–53.

[19] Wray LD, Stone ER (2005) The role of self-esteem and anxiety in decision making for self versus others in relationships. Journal of Behavioural Decision Making 182: 125–144.

[20] Simpkin AL, Robertson LC, Barber VS, Young JD (2009) Modifiable factors influencing relatives’ decision to offer organ donation: Systematic review. British Medical Journal 338: 991. 10.1136/bmj.b991 PMC2671586 19383730

[21] Jones B, Rachlin H (2006) Social discounting. Psychological Science 17: 283–286. 16623683 10.1111/j.1467-9280.2006.01699.x

[22] Rachlin H, Jones BA (2008) Altruism among relatives and non-relatives. Behavioural Processes 79: 120–123. 18625292 10.1016/j.beproc.2008.06.002 PMC2561243

[23] Wright S (1921) Systems of mating. Genetics 6: 111–178. 17245958 10.1093/genetics/6.2.111 PMC1200501

[24] Krebs JR, Davies NB (1993) An introduction to behavioural ecology, 3rd edition. New York: Blackwell Scientific.

[25] Peirce JW (2008) Generating stimuli for neuroscience using PsychoPy. Neuroinform 2: 10 doi: 10.3389/neuro.11.010.2008. 10.3389/neuro.11.010.2008 PMC2636899 19198666

[26] Stillwell DJ, Tunney RJ (2012) Effects of measurement methods on the relationship between smoking and delay reward discounting. Addiction 107(5): 1003–1012. 22126134 10.1111/j.1360-0443.2011.03742.x

[27] Rachlin H (2000) The science of self-control. Cambridge, MA: Harvard University Press.

[28] Mazur JE (1987) An adjusting procedure for studying delayed reinforcement. In: Commons ML, Mazur JE, Nevin JA, Rachlin H, editors. Quantitative analyses of behavior, Vol. 5. The effect of delay and of intervening events on reinforcement value. Hillsdale, NJ: Erlbaum. 55–73.

[29] Shalowitz DI, Garret-Mayer E, Wendler D (2006) The accuracy of surrogate decision makers: A systematic review. Archives of Internal Medicine 166: 493–497. 16534034 10.1001/archinte.166.5.493

